# Motor skills influence social function through health-related fitness in children with autism: a cross-sectional study

**DOI:** 10.3389/fpubh.2026.1758323

**Published:** 2026-02-23

**Authors:** Hui Shen, Zixuan Zou, Shuqi Jia, Qiang Wang, Zehui He, Mengsi Chen, Zhenyu Zhang

**Affiliations:** 1 Department of Physical Education, Ma'anshan University, Ma’anshan, Anhui, China; 2 School of Education Science, Nanjing Normal University of Special Education, Nanjing, China; 3 School of Athletic Performance, Shanghai University of Sport, Shanghai, China; 4 School of Athletic Performance, Guangdong Vocational Institute of Sport, Guangzhou, China; 5 School of Exercise and Health, Shanghai University of Sport, Shanghai, China; 6 School of Physical Education and Health, Shanghai Lixin University of Accounting and Finance, Shanghai, China

**Keywords:** autism spectrum disorder, fundamental motor skills, health-related fitness, mediation, social functioning, structural equation modeling

## Abstract

**Objective:**

To examine whether health-related physical fitness mediates the association between fundamental motor skills (FMS) and social functioning in school-age children with autism spectrum disorder (ASD).

**Methods:**

A total of 117 school-age children with ASD were recruited from special education schools. FMS were assessed using the Test of Gross Motor Development–3 (TGMD-3). Social functioning was evaluated using the Social Responsiveness Scale–2 (SRS-2), with higher scores indicating greater social impairment. Health-related physical fitness was assessed via body mass index, flexibility (sit-and-reach), muscular strength (handgrip), muscular power (standing long jump), balance (one-leg stance), and vital capacity. Pearson correlation analyses and structural equation modeling with bias-corrected bootstrapping (5,000 resamples) were conducted to test mediation effects.

**Results:**

Higher FMS scores were associated with lower SRS-2 scores, indicating better social functioning (*r* = −0.312, *p* < 0.001). Several health-related physical fitness components, particularly handgrip strength and flexibility, were significantly associated with both FMS and social functioning. Structural equation modeling demonstrated a full mediation effect, whereby health-related physical fitness significantly transmitted the association between FMS and social functioning (indirect effect B = −2.111, 95% CI [−3.576, −1.189]), while the direct effect was not significant (B = −0.095, 95% CI [−1.193, 1.274]). Model fit indices indicated an excellent fit.

**Conclusion:**

Health-related physical fitness, especially muscular strength and flexibility, appears to be a key mechanism linking motor skill proficiency to social functioning in children with ASD. Interventions that integrate strength and mobility training with motor-skill practice may enhance social outcomes in this population.

## Introduction

Autism spectrum disorder (ASD) is characterized by persistent social communication difficulties and restricted, repetitive behaviors, affecting children’s ability to engage in peer interactions and participate in daily life. Social impairments are common and significantly influence school adaptation, interpersonal development, and long-term independence (1–3). Motor difficulties frequently co-occur with ASD, including deficits in balance, coordination, and motor planning, which may further interfere with opportunities for social engagement (4–6).

Fundamental motor skills (FMS)—including locomotor skills and object-control skills—are foundational movement capacities supporting participation in physical and social activities (7). Children with ASD often exhibit delayed or impaired FMS, which can reduce participation in play, limit peer interaction, and hinder the development of social skills (8, 9). Although prior studies have shown associations between motor ability and social functioning (10), the underlying mechanism linking FMS to social development in ASD remains insufficiently understood.

Health-related physical fitness represents a potential mechanistic bridge linking fundamental motor skills (FMS) to social functioning. Fitness components such as muscular strength, flexibility, and cardiorespiratory capacity contribute to movement efficiency, physical participation, and behavioral self-regulation (11–13). Although prior research has documented associations between motor competence and social functioning in ASD, the underlying mechanisms of this association remain insufficiently understood (14). Previous studies further report that children with ASD typically have lower fitness levels compared with typically developing peers (15), and better physical fitness is associated with improved adaptive behavior and participation (16). However, few studies have explicitly examined whether physical fitness mediates the relationship between motor proficiency and social functioning in ASD, leaving gaps in mechanistic understanding.

Emerging theories suggest that motor competence may support autonomy, perceptual-motor coupling, and social motivation, while physical fitness may enhance endurance, postural control, and coordination—key capacities for interactive play and communication (17, 18). Therefore, fitness may be a modifiable pathway through which motor-skill development translates into social gains.

This study aimed to investigate whether health-related physical fitness mediates the association between FMS and social functioning in school-age children with ASD. We hypothesized that: (1) higher FMS would be associated with better social functioning; (2) FMS would be positively associated with fitness; and (3) fitness would mediate the association between FMS and social functioning, with muscular strength and flexibility showing particularly strong contributions.

## Methods

### Participants

A total of 117 children diagnosed with autism spectrum disorder (ASD) were recruited from two special education schools in multiple cities within the Pearl River Delta region of China, including Guangzhou, Shenzhen, Zhuhai, Foshan, and Zhongshan, using a convenience sampling strategy. During the recruitment process of children with moderate ASD, a small number of female children were initially identified. To control for potential sex-related effects and given the limited number of female participants, female children were excluded from the final analysis. Consequently, the final sample consisted of 117 male children with ASD.

Inclusion criteria were: (1) a clinical diagnosis of ASD confirmed by a licensed clinician based on DSM-5 criteria, and meeting the diagnostic criteria for moderate autism spectrum disorder according to the Chinese Classification and Diagnostic Criteria of Mental Disorders, Third Edition (CCMD-3), with behavioral manifestations assessed as moderate impairment and classified as Level II (requiring substantial support); (2) age between 6 and 12 years; (3) ability to follow simple verbal instructions and complete physical assessments; and (4) informed consent obtained from parents or legal guardians. Exclusion criteria included: (1) comorbid neurological or severe physical impairments that could affect motor performance or participation (e.g., cerebral palsy, muscular dystrophy); (2) recent musculoskeletal injury within the past 3 months; and (3) acute illness on the day of testing.

This study complied with the ethical requirements of the latest version of the Declaration of Helsinki and was reviewed by the Ethics Committee of Shanghai University of Sport (102772020RT061). This study recruited participants and conducted tests from March 2020 to December 2020.

### Study design and procedure

This cross-sectional study was conducted in designated assessment rooms within the schools. Assessments were administered individually by trained physical education and rehabilitation instructors who completed standardized training in test administration prior to data collection. Participants completed motor skill testing, physical fitness assessments, and questionnaires measuring social functioning on the same day, with scheduled rest breaks to minimize fatigue and ensure optimal participation.

### Measurement index and test methods

#### Measurement index

1. Health-related physical fitness assessment: Health-related fitness indicators were selected based on the National Student Physical Health Standard and the Brockport Physical Fitness Test (BPFT) (19). The assessments included measurements of body mass index (BMI), flexibility (sit-and-reach), vital capacity, muscular fitness (handgrip strength and standing long jump), and balance (one-leg stance with eyes open) (Table 1).
2. Fundamental Motor Skills Assessment: Fundamental motor skills were evaluated using the Test of Gross Motor Development–Third Edition (TGMD-3), developed by Dale A. Ulrich at the University of Michigan, which is widely used for assessing motor competence in children aged 3–10 years (20). The TGMD-3 consists of two subtests—locomotor skills and ball skills—with 13 test items in total. The locomotor subtest includes six items: run, gallop, hop, skip, horizontal jump, and slide. The ball-skills subtest includes seven items: two-hand strike of a stationary ball, one-hand forehand strike of a self-bounced stationary ball, stationary dribble, two-hand catch, kick, overhand throw, and underhand throw. Each skill contains 3–5 performance criteria, scored as 1 (criterion met) or 0 (criterion not met). Each item was performed twice, and the sum of both trials represented the final item score. Total TGMD-3 scores ranged from 0 to 100 points, with the locomotor and ball-skills subtests contributing 46 and 54 points, respectively. Before testing, demographic information (e.g., sex, grade) and routine physical characteristics (e.g., weight, dominant hand/foot) were collected. Assessments were administered jointly by trained graduate students in physical education and experienced special-education physical educators. For each task, the examiner demonstrated the movement 2–3 times, followed by two practice trials and two formal trials by each child. All sessions were video-recorded. Two independent raters scored each video, and the mean of the two ratings was used as the final score. Discrepancies were resolved by a third rater to ensure scoring reliability and objectivity.
3. Social Skill Assessment: Social functioning was assessed using the Social Responsiveness Scale–Second Edition (SRS-2), a validated measure widely used to quantify social impairment in children with autism spectrum disorder (21). The SRS-2 evaluates multiple domains, including social communication, social motivation, social awareness, and restricted or repetitive behaviors. The scale demonstrates excellent internal consistency, with a Cronbach’s alpha of 0.946 for the total score and 0.828–0.936 across subscales.

**Table 1:** Health-related physical fitness test items, instruments, and procedures.

Test item	Instrument	Testing procedure
Body mass index (BMI)	Body composition analyzer (OMRON HBF-306)	Participants removed shoes and stood upright on the device. BMI was automatically calculated.
Flexibility (Sit-and-reach)	Adjustable flexibility tester (DELI FT303)	Participants removed their shoes and sat on a mat, with legs fully extended and hands placed on the slider. After two familiarization trials, participants slowly leaned forward and pushed the slider as far as possible without bending knees. Best value recorded (cm).
Vital capacity	Portable spirometer (Norenka TRX; accuracy 1 mL)	Participants inhaled maximally and then exhaled forcefully and continuously into the mouthpiece without secondary inhalation. Three trials were performed, and the highest value was recorded (mL).
Muscular fitness	Handgrip dynamometer (Condo EH106; accuracy 0.1 kg) and standing-long-jump mat (2.5 m × 0.9 m rubber mat; accuracy 1 cm)	Grip strength: Participants squeezed the dynamometer with their dominant hand as hard as possible. Three trials performed; best value recorded (kg). Standing long jump: Participants jumped forward from a standing start; arm swing allowed. Three trials performed; best value recorded (cm).
Balance	One-leg stance test	Participants stood on their dominant leg with hands placed on the waist and the non-support foot touching the knee of the stance leg. After the “start” command, participants maintained balance as long as possible. Time stopped when the lifted foot touched the ground or posture changed. Three trials performed; best time recorded (s).

### Procedure

All procedures adhered to the ethical standards of the Declaration of Helsinki and were approved by the Ethics Committee of Shanghai University of Sport (Approval No. 102772020RT061). Written informed consent was obtained from the parents or legal guardians of all participants prior to data collection.

**Figure 1:**
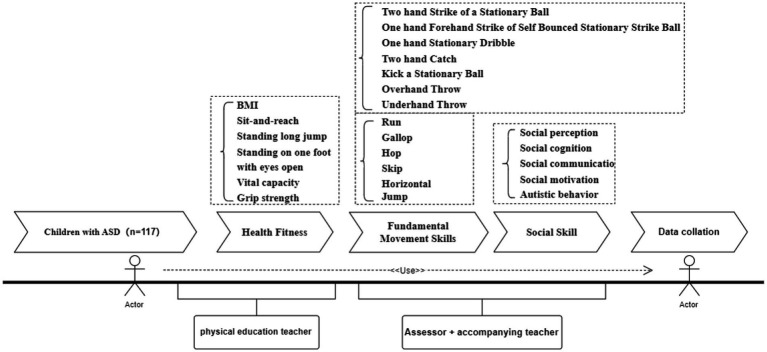
Test process.

Data were collected between September and December 2020 in special education schools. Testing was conducted during non-instructional hours (3:30–5:30 p.m.) to avoid interference with regular learning activities. Class teachers assisted in organizing the participants and maintaining testing order. The assessment sequence followed a standardized protocol. First, demographic and routine physiological information (e.g., age, sex, height, weight, dominant hand and foot) was recorded. Participants then completed the fundamental movement skill assessment using the Test of Gross Motor Development-3 (TGMD-3), followed by the health-related physical fitness tests, and finally the evaluation of social skills using the Social Responsiveness Scale-2 (SRS-2). All tests were administered by trained postgraduate students majoring in physical education and experienced physical education teachers from special education schools. Prior to formal testing, children received familiarization demonstrations. MOVEMENT performance was recorded using a digital camera, and two independent raters scored the videos. Discrepancies were reviewed by a third trained assessor to ensure scoring accuracy and inter-rater reliability. The complete testing workflow is illustrated in Figure 1.

### Statistical methods

The distribution of all variables was examined using frequency histograms. Continuous data that followed a normal or approximately normal distribution were presented as mean ± standard deviation (M ± SD). For group comparisons, one-way ANOVA was applied when the assumption of homogeneity of variance was met, whereas the Brown–Forsythe test was used when this assumption was violated. Non-normally distributed data were expressed as median and interquartile range 𝑀(𝑃25, 𝑃75), and the Kruskal–Wallis test was used for non-parametric group comparisons. Pearson correlation analysis was employed to examine the associations among FMS, social skills, and multiple dimensions of health-related fitness. All tests were two-tailed, and a significance level of *α* = 0.05 was adopted. Statistical significance was defined as *p* < 0.05, *p* < 0.01, and *p* < 0.001. To assess potential common-method bias, Harman’s single-factor test was conducted. Structural equation modeling (SEM) was performed to evaluate the mediating effect of health-related fitness on the association between FMS and social skills. All variables were standardized prior to modeling. Model fit was assessed using RMR, RMSEA, GFI, NFI, and CFI indices. Bias-corrected bootstrapping (5,000 resamples) was used to estimate path coefficients and their 95% confidence intervals. A mediating effect was considered significant when the confidence interval did not include zero. All analyses were conducted using SPSS Statistics version 24.0 and AMOS version 24.0. Results were reported to three decimal places, with percentages presented to two decimal places.

## Results

### Differences in health-related fitness and social skills across fundamental motor skill levels

As shown in Table 2, a total of 117 children with ASD in grades 1–6 were included in the analysis. The mean body mass index was 18.21 ± 3.80 kg/m^2^, sit-and-reach flexibility averaged −10.43 ± 5.99 cm, standing long jump distance was 69.82 ± 15.53 cm, one-leg stance time with eyes open averaged 5.79 ± 3.04 s, vital capacity was 521.86 ± 147.02 mL, and handgrip strength was 7.06 ± 2.26 kg. No significant differences were observed among different FMS levels for vital capacity, one-leg stance time, or sit-and-reach performance (*p* > 0.05). However, significant differences were found in social skill scores (142.88 ± 10.90) across FMS levels (*p* < 0.05). Additionally, FMS performance significantly differed by school grade (*p* < 0.05), suggesting developmental variation in motor ability across age groups.

**Table 2:** Differences in grades, health fitness and social skill of children with ASD among different levels of fundamental movement skills.

Variables	Total (*n* = 117)	Low level (*n* = 42)	Medium-low level (*n* = 57)	Medium-high level (*n* = 18)	Difference test
Grade (1 ~ 6)	3(2, 5)	2(1, 3)	4 (2, 5)	5 (3, 5)	χ^2^ = 21.670, *p* < 0.001
BMI/(kg·m^−2^)	18.206 ± 3.804	17.022 ± 2.621	18.298 ± 3.463	20.678 ± 5.717	*F* = 6.397, *p* = 0.002
Sit-and-reach (cm)	−10.43 ± 5.992	−8.81 ± 0.984	−11.09 ± 0.738	−12.11 ± 1.372	*F* = 2.662, *p* = 0.074
Standing long jump	69.82 ± 15.528	65.55 ± 2.523	70.02 ± 1.459	79.17 ± 5.071	*F* = 5.207, *p* = 0.007
Standing on one foot with eyes open	5.79 ± 3.039	5.90 ± 0.533	5.91 ± 0.287	5.17 ± 1.007	*F* = 0.450, *p* = 0.639
Vital capacity (ml)	521.86 ± 147.023	498.71 ± 167.574	506.04 ± 136.454	567.50 ± 120.926	*F* = 1.512, *p* = 0.225
Grip strength (kg)	7.061 ± 2.264	5.995 ± 1.743	7.440 ± 2.375	8.344 ± 2.010	*F* = 9.581, *p* < 0.001
Social skill	142.88 ± 10.903	147.63 ± 11.844	140.53 ± 9.920	139.28 ± 7.797	*F* = 6.919, *p* = 0.001

### The relationship between fundamental movement skills, health fitness and social skill in children with ASD

#### Relationship between fundamental motor skills and social skills

**Figure 2:**
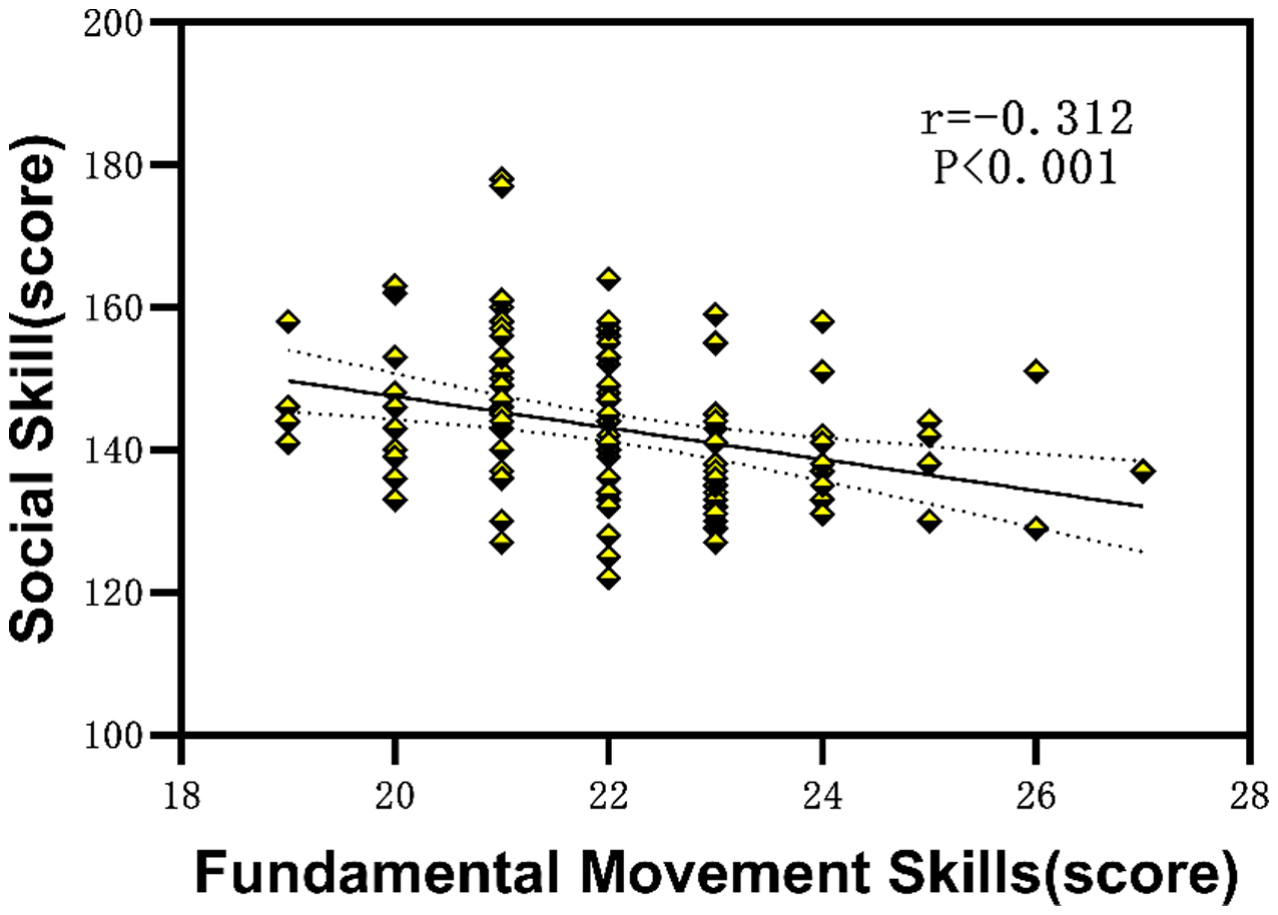
The relationship between fundamental movement skills and social skill in children with ASD.

Linear regression analysis was conducted to examine the association between fundamental motor skills and social functioning. As shown in Figure 2, a significant negative correlation was observed between FMS scores and social impairment scores (*r* = −0.312, *R*^2^ = 0.097, *p* < 0.001). Children with higher FMS demonstrated lower levels of social impairment, indicating better social functioning.

#### Relationship between health-related fitness and social skills

**Figure 3:**
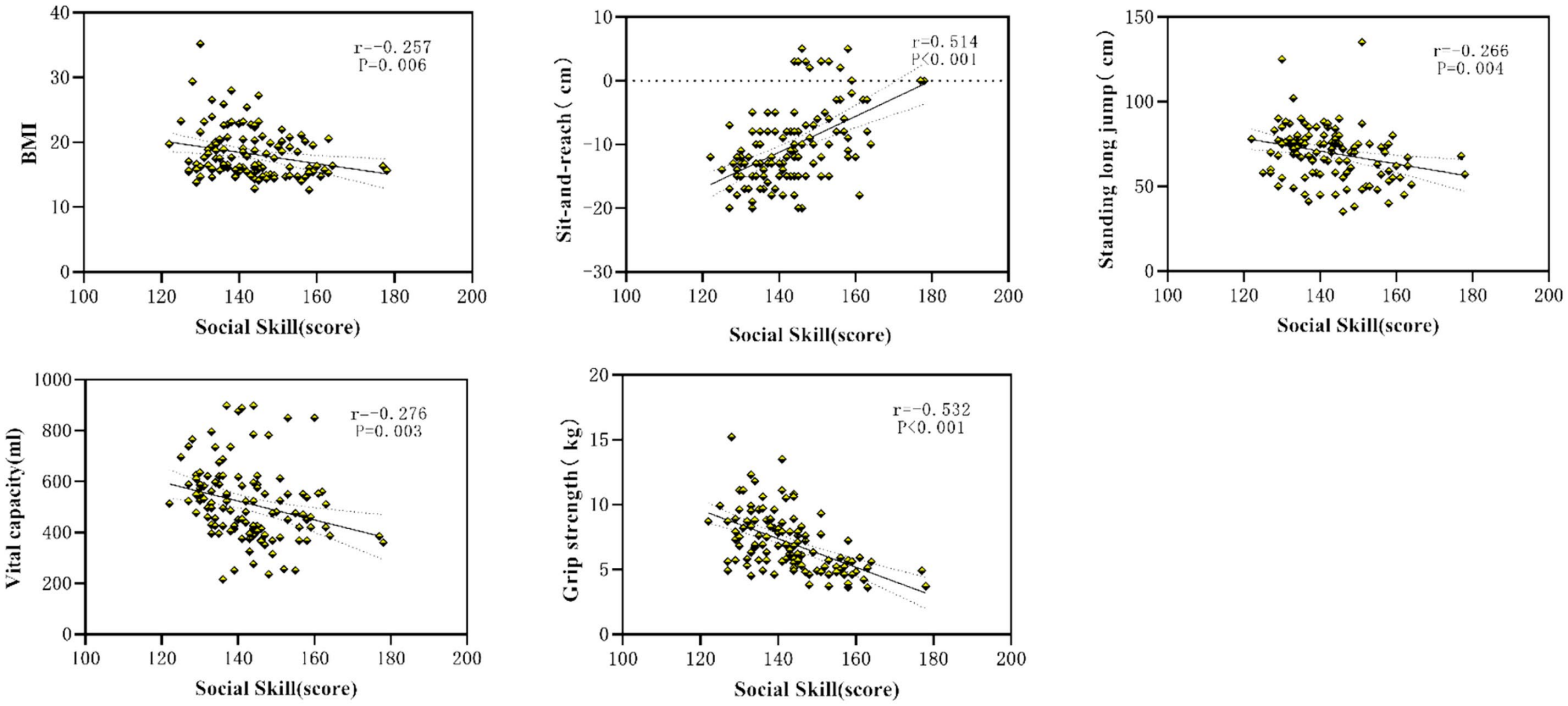
The indexes of health fitness that were sensitive to social skill.

Pearson correlation analysis was performed to explore associations between components of health-related fitness and social functioning. As shown in Table 3, BMI, standing long jump, vital capacity, and handgrip strength were significantly negatively correlated with SRS-2 scores (all *p* < 0.01), while sit-and-reach flexibility was significantly positively correlated with SRS-2 scores (*p* < 0.01). One-leg stance performance was not significantly associated with SRS-2 scores. These findings suggest that multiple fitness indicators, particularly muscular fitness and flexibility, are linked to social functioning in children with ASD (Figure 3).

**Table 3:** Correlation coefficients between the dimensions of health fitness and SRS-2 scores.

Factor	Statistic	BIM	Sit-and-reach	Standing long jump	Standing on one foot with eyes open	Vital capacity	Grip strength
Social skill	Correlation coefficient	−0.251**	0.513**	−0.266**	0.001	−0.276**	−0.532**
	*P*	0.006	<0.001	0.004	0.995	0.003	<0.001

* and ** indicated *P* < 0.05 and *P* < 0.01 respectively, the same below.

#### Relationship between fundamental motor skills and health-related fitness

**Figure 4:**
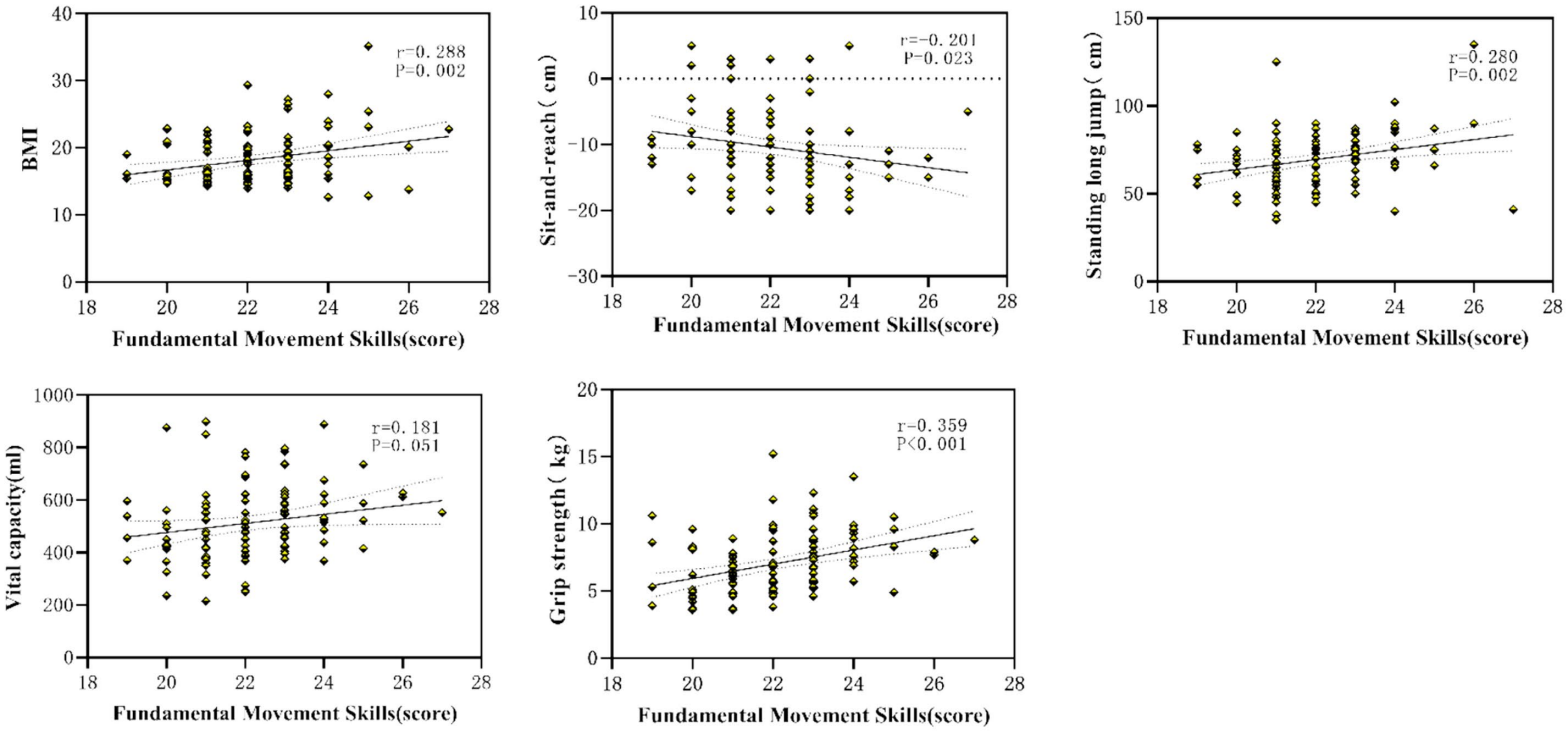
The indexes of health fitness that were sensitive to fundamental movement skills.

To further identify fitness indicators most sensitive to FMS, Pearson correlation analyses were conducted. BMI, standing long jump, vital capacity, and handgrip strength were significantly positively correlated with FMS level (all *p* < 0.05), while sit-and-reach flexibility was negatively correlated with FMS. One-leg stance performance was not significantly associated with FMS. Together with Figure 4, these results indicate that BMI, flexibility, muscular strength, power, and vital capacity represent fitness pathways linking motor competence to social functioning (Table 4).

**Table 4:** Correlations between fundamental movement skills and specific indexes of health fitness in children with ASD.

Factor	Statistic	BIM	Sit-and-reach	Standing long jump	Standing on one foot with eyes open	Vital capacity	Grip strength
Fundamental movement skills	Correlation coefficient	0.288**	−0.201*	0.280**	0.020	0.181	0.359**
	P	0.002	0.030	0.002	0.883	0.051	<0.001

### The construction and verification of the structural relationship among the indexes of fundamental movement skills, health fitness and social skill of children with ASD

**Figure 5:**
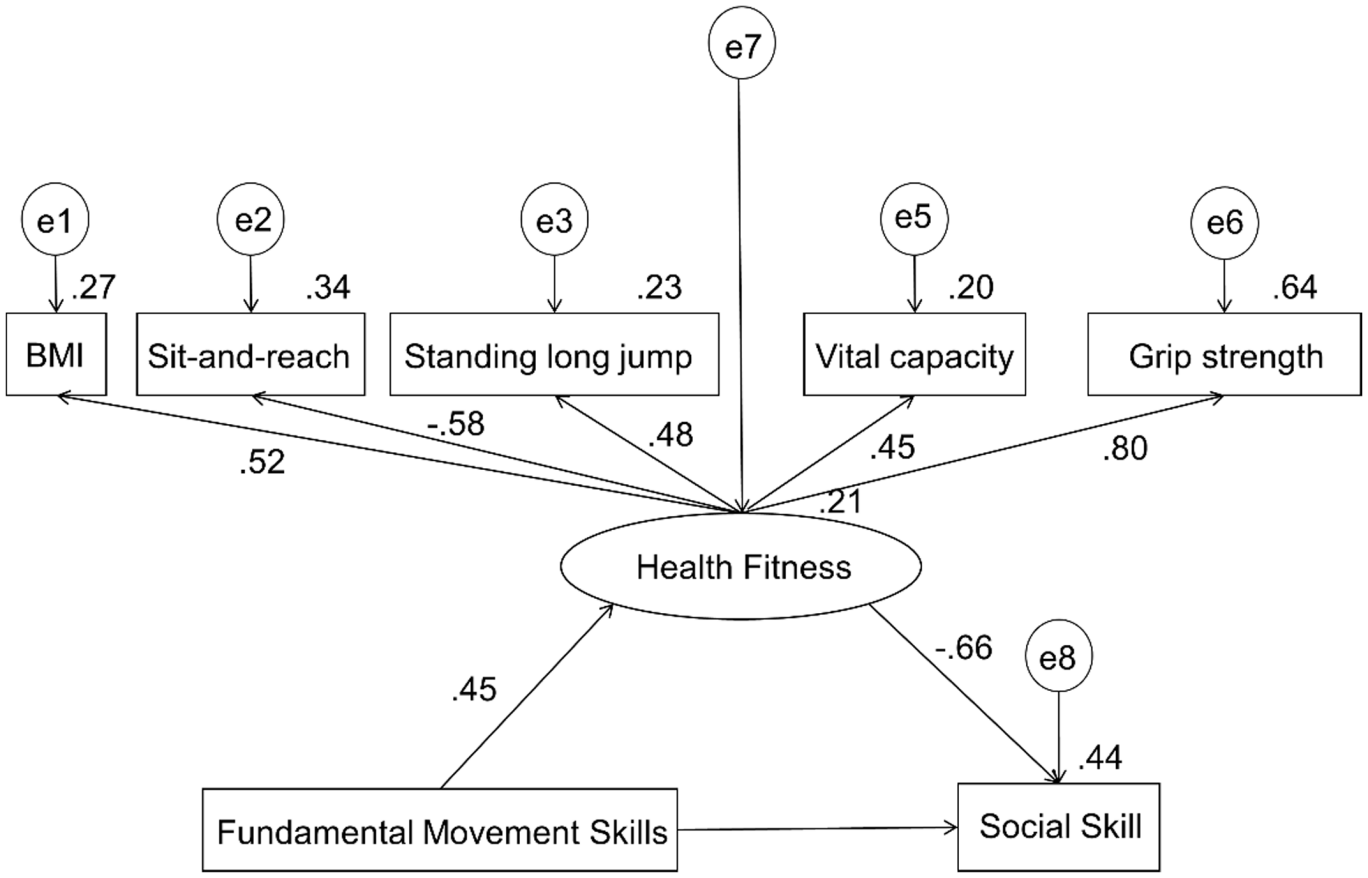
The structural relationship of fundamental movement skills, social skill, and specific indexes of health fitness in children with ASD.

Based on the correlation between fundamental movement skills, health fitness and social skill of children with moderate autism, Harman’s single-factor test was used to conduct exploratory factor analysis to examine the specific indexes that were significantly correlated with the scores of fundamental movement skills, health fitness and social skill. Two factors with eigenvalues greater than 1 were extracted, and the variance explained by the first factor was 37.01%, which was below the critical threshold of 40%, indicating that common method bias was within an acceptable range. Next, a structural equation model was established with fundamental movement skills as the independent variable, social skill as the dependent variable, and health fitness as the mediator. The model fit indices were as follows: CMIN/df = 1.233, RMSEA = 0.045, GFI = 0.961, NFI = 0.910, and CFI = 0.981. In addition, the standardized root mean square residual (SRMR) was below the recommended threshold of 0.08, indicating an acceptable model fit. Overall, these indices met commonly accepted criteria (CMIN/df < 3, RMSEA < 0.08, GFI, NFI, and CFI > 0.90), suggesting that the structural equation model demonstrated a good fit to the data. The path analysis is shown in Figure 5, and the results of the mediation effect test are presented in Table 5. The results showed that fundamental movement skills had a direct effect of 4.3% on social skill (B = −0.095, 95% CI = −1.193 to 1.274), indicating that higher levels of fundamental movement skills were associated with lower social skill scores; however, this direct effect was not statistically significant. Furthermore, health fitness, as a latent variable, accounted for 95.7% of the total indirect effect (B = −2.111, 95% CI = −3.576 to −1.189). The path coefficients for BMI, sit-and-reach, standing long jump, vital capacity, and grip strength were all significant, with grip strength and sit-and-reach showing relatively stronger loadings. These results indicate that health fitness mediates the relationship between fundamental movement skills and social skill, with grip strength and flexibility exerting greater influence.

**Table 5:** The results of bootstrap test for the mediator effect.

Effect category	B	95%CI	*P*	SE	Effect proportion/%
Total effect	−2.206	[3.364, −1.121]	<0.001	0.567	100
Direct effect	−0.095	[−1.193, 1.274]	0.897	0.621	4.3
Mediator effect	−2.111	[−3.576, −1.189]	<0.001	0.608	95.7

## Discussion

The present study found that children with ASD with better fundamental movement skills exhibited higher levels of physical fitness and social skills. Significant correlations were observed among physical fitness, FMS, and social skills. Specifically, muscular fitness and flexibility showed moderate correlations with social skills, whereas muscular fitness, BMI, and flexibility demonstrated moderate-to-weak correlations with FMS. Furthermore, health-related physical fitness fully mediated the association between FMS and social skills, and the strength of mediation varied across different fitness indicators.

These results suggest that children with ASD who demonstrate stronger motor skills tend to have more favorable social and fitness outcomes. Improvements in physical fitness may enhance children’s adaptive behavior and facilitate the emergence of positive social behaviors, forming a virtuous cycle of participation and engagement (22, 23). Enhanced fitness may reduce motor dysfunction, increase participation opportunities, and provide a foundation for exercise-based interventions that support executive function improvements and promote neuroplastic changes in executive control networks (24, 25).

Importantly, the study confirmed that physical fitness fully mediates the relationship between FMS and social functioning, with muscular fitness and flexibility emerging as key mediating pathways. This underscores the interdependence between basic motor competence and fitness development in promoting social gains among children with ASD.

First, the development of FMS requires the participation of physical fitness components, including effective coordination and manipulation of limb movements, power production, and sustained neuromuscular activation (26). Physical activity influences not only gross motor skills but also contributes to improved physical fitness, indirectly fostering better social functioning (27). Regular participation in activities such as walking, running, jumping, and climbing enhances muscular endurance (25). Continued engagement in physical activity provides sensory-motor experience that shapes motor development trajectories (28), and repeated muscular activation contributes to higher endurance and cardio-respiratory capacity (11).

Second, muscular strength plays a vital role in the social development of children with ASD. Delayed motor milestones are common in ASD (29). Handgrip strength is particularly important for early environmental exploration, object manipulation, and initiating social interactions (30). Skills like reaching and grasping promote joint engagement with objects and caregivers, increasing opportunities for communicative gestures and shared attention (25). Furthermore, lower-limb muscle strength supports independent walking, which is associated with perceptual, cognitive, and social developmental changes. Upright mobility expands the child’s visual field, enabling improved facial recognition and environmental awareness, thereby promoting social interaction (31, 32).

Third, flexibility also appears to play a meaningful role in social engagement in children with ASD. It should be noted that higher SRS-2 scores indicate greater social impairment rather than better social functioning. In contrast to typically developing patterns, flexibility in children with ASD did not improve with age or motor skill level; instead, a decline was observed. This pattern may reflect atypical neuromuscular control, reduced postural stability, or altered muscle tone rather than functional motor proficiency. Excessive flexibility or joint laxity in ASD has been linked to sensory modulation difficulties and poor motor coordination, which may negatively affect daily functioning and participation (33). Social opportunities often arise during cooperative play (34); however, children with ASD may miss these opportunities due to motor slowness, clumsiness, or reduced postural control, limiting their ability to form and maintain peer relationships (35). Reduced flexibility-related motor control may further affect gait performance (36), and over time, diminished social participation may contribute to delays in social communication development (37, 38).

## Limitations and future directions

This study adopted a cross-sectional design, limiting causal inference. This study adopted a cross-sectional design, limiting causal inference. In addition, developmental covariates such as age, sex, and ASD severity were not included in the SEM due to sample homogeneity (all participants were male children with moderate ASD) and structural constraints within the special education system; future studies with more heterogeneous samples should incorporate these variables. Future research should employ longitudinal or intervention designs to verify the mediating role of fitness in the relationship between FMS and social functioning. Additionally, the physical fitness indicators used in this study reflected the developmental characteristics of school-age children with ASD; future work should expand assessment instruments across different developmental stages. Finally, the sample was restricted to school-age children in special-education settings; subsequent research should include preschool populations and examine how FMS-based interventions influence longitudinal motor and social development, supporting evidence-based motor prescriptions in clinical practice.

## Conclusion

This study demonstrated that health-related physical fitness fully mediates the association between fundamental motor skills and social functioning in children with autism spectrum disorder. Specifically, muscular strength and flexibility emerged as key fitness components contributing to this mediating pathway. These findings suggest that motor skill development alone may not be sufficient to enhance social functioning unless accompanied by improvements in physical fitness. Integrating strength and flexibility training with motor skill interventions may therefore represent an effective approach to promote both physical competence and social participation in children with autism. Future longitudinal and intervention studies are warranted to further validate the causal direction of these relationships and to explore optimal exercise prescriptions for this population.

## Data Availability

The raw data supporting the conclusions of this article will be made available by the authors, without undue reservation.

## References

[1] Sharma SR Gonda X Tarazi FI. Autism spectrum disorder: classification, diagnosis and therapy. Pharmacol Ther. (2018) 190:91–104. doi: 10.1016/j.pharmthera.2018.05.007, 29763648

[2] Salari N Rasoulpoor S Rasoulpoor S Shohaimi S Jafarpour S Abdoli N . The global prevalence of autism spectrum disorder: a comprehensive systematic review and meta-analysis. Ital J Pediatr. (2022) 48:112. doi: 10.1186/s13052-022-01310-w, 35804408 PMC9270782

[3] Wang F Lu L Wang SB Zhang L Ng CH Ungvari GS . The prevalence of autism spectrum disorders in China: a comprehensive meta-analysis. Int J Biol Sci. (2018) 14:717–25. doi: 10.7150/ijbs.24063, 29910682 PMC6001678

[4] Ma X Cai Y Chen S Li K Zhuang P. Relationship between fundamental motor skills and sedentary behavior in preschool children aged 3–6 years. J Phys Educ. (2019) 26:123–8. doi: 10.16237/j.cnki.cn44-1404/g8.20190606.008

[5] Craig F Crippa A Ruggiero M Rizzato V Russo L Fanizza I . Characterization of autism spectrum disorder subtypes based on the relationship between motor skills and social communication abilities. Hum Mov Sci. (2021) 77:102802. doi: 10.1016/j.humov.2021.102802 33894651

[6] Zhang L Li H Wang H Hu S Wang Z. Correlation between fundamental motor skills and physical fitness in preschool children. Zhongguo Xue Xiao Wei Sheng. (2020) 41:554–7. doi: 10.16835/j.cnki.1000-9817.2020.04.020

[7] Wang H Hu S Li Y Zhen Y. Canonical correlation analysis of motor skills and physical fitness in preschool children. China Sport Sci Technol. (2019) 55:46–51. doi: 10.16470/j.csst.2019018

[8] Chen W Zhu X Zhang H. Progress in motor function assessment and intervention for children with ASD. China Sport Sci Technol. (2022) 58:3–9. doi: 10.16470/j.csst.2021008

[9] Xu J Cai Y Ma X Wang J Liu S Chen S. Review and insights on relationships among fundamental movement skills, perceptual-motor ability, and physical activity in children and adolescents. J Cap Univ Phys Educ Sports. (2021) 33:686–96. doi: 10.14036/j.cnki.cn11-4513.2021.06.014

[10] Kong C Chen A Ludyga S Herold F Healy S Zhao M . Associations between meeting 24-hour movement guidelines and quality of life among children and adolescents with autism spectrum disorder. J Sport Health Sci. (2023) 12:73–86. doi: 10.1016/j.jshs.2022.08.003, 36029958 PMC9923433

[11] Li Y Sun L Jiang W Yang S Ren Y Wang H. Relationship between gross motor development and physical fitness in children aged 3–5 years. Zhongguo Xue Xiao Wei Sheng. (2019) 40:1194–9. doi: 10.16835/j.cnki.1000-9817.2019.08.020

[12] Bremer E Crozier M Lloyd M. A systematic review of behavioral outcomes following exercise interventions for children and youth with autism. Autism. (2016) 20:899–915. doi: 10.1177/1362361315616002, 26823546

[13] Pan CY Tsai CL Chu CH. Fundamental movement skills in children with ASD and ADHD. J Autism Dev Disord. (2009) 39:1694–705. doi: 10.1007/s10803-009-0813-5, 19588236

[14] Sefen JAN Al-Salmi S Shaikh Z AlMulhem JT Rajab B Fredericks S. Beneficial use and potential effectiveness of physical activity in managing ASD. Front Behav Neurosci. (2020) 14:587560. doi: 10.3389/fnbeh.2020.587560, 33192368 PMC7642468

[15] Healy S Nacario A Braithwaite R Hopper C. Effect of physical activity interventions on youth with ASD: a meta-analysis. Autism Res. (2018) 11:818–33. doi: 10.1002/aur.1955, 29693781

[16] Westendorp M Houwen S Hartman E Visscher C. Gross motor skills and sports participation in children with intellectual disabilities. Res Dev Disabil. (2011) 32:1147–53. doi: 10.1016/j.ridd.2011.01.009, 21310587

[17] Hill PJ McNarry MA Mackintosh KA Murray MA Pesce C Valentini NC . Longitudinal associations between motor competence and cognitive and socio-emotional outcomes. Sports Med. (2024) 54:375–427. doi: 10.1007/s40279-023-01939-5, 37989831 PMC10933160

[18] Lopes L Santos R Coelho ESM Draper C Mota J Jidovtseff B . A Narrative Review of Motor Competence in Children and Adolescents: What We Know and What We Need to Find Out. Int J Environ Res Public Health. (2020) 18:18. doi: 10.3390/ijerph18010018 33375134 PMC7792958

[19] Chen H. Research and implications of U.S. health-related fitness standards for youth with disabilities. J Shanghai Univ Sport. (2017) 41:23–34. doi: 10.16099/j.sus.2017.03.004

[20] Allen KA Bredero B Van Damme T Ulrich DA Simons J. Test of Gross Motor Development-3 (TGMD-3) with the Use of Visual Supports for Children with Autism Spectrum Disorder: Validity and Reliability. J Autism Dev Disord. (2017) 47:813–33. doi: 10.1007/s10803-016-3005-0, 28091840

[21] Chen L Shu Y Liu X Ji Y Dai Y Wu D. Applicability of brief SRS in Chinese children with autism. J Educ Biol. (2019) 7:80–5. doi: 10.3969/j.issn.2095-4301

[22] Jin C Cao M Gu T Li X Jing J. Relationship between motor function and core symptoms in children with ASD. Zhongguo Xue Xiao Wei Sheng. (2023) 44:176–85. doi: 10.16835/j.cnki.1000-9817.2023.02.004

[23] Zhang J Wang S Lin Q. Exercise intervention research on autism: a review. Zhongguo Yundong Yixue Zazhi. (2017) 36:552–7. doi: 10.16038/j.1000-6710.2017.06.016

[24] Song B Wang Y Wang D Bai K. ICF-CY-based review on physical activity participation in children and adolescents with ASD. Zhongguo Kangfu Lilun Yu Shijian. (2022) 28:1309–17. doi: 10.3969/j.issn.1006-9771.2022.11.010

[25] Bradshaw J Klaiman C Gillespie S Brane N Lewis M Saulnier C. Walking ability is associated with social communication skills in infants at high risk for autism spectrum disorder. Infancy. (2018) 23:674–91. doi: 10.1111/infa.12242, 30271278 PMC6159946

[26] Wang L Wang Z Wang H. Neural mechanisms of motor development disorders in children with ASD. Adv Psychol Sci. (2021) 29:1239–50. doi: 10.3724/SP.J.1042.2021.01239

[27] Green AE Kenworthy L Gallagher NM Antezana L Mosner MG Krieg S . Social analogical reasoning in children with ASD. Autism. (2017) 21:403–11. doi: 10.1177/1362361316644728, 27178998 PMC6085745

[28] Marraffa C. Social communication in ASD not improved by theory-of-mind intervention. J Paediatr Child Health. (2016) 52:461–3. doi: 10.1111/jpc.13178, 27145512

[29] Li M Wei L Gu Q. Rehabilitation effect of fine-motor learning in children with ASD. Chin J Rehabil Theory Pract. (2016) 22:1314–7. doi: 10.3969/j.issn.1006-9771

[30] Gao Y Chen A Wei G. Motor imitation deficit in ASD: behavioral and brain perspectives. Sci Technol Rev. (2022) 40:97–109. doi: 10.3981/j.issn.1000-7857

[31] Ji H Wang L Jiang Y. Atypical social attention and neural mechanism in ASD. Chin Sci Bull. (2018) 63:1428–37. doi: 10.1360/N972017-01133

[32] Chen X Chen L. Head rotation interpersonal synchrony deficit in ASD children. Chin Sci Bull. (2022) 67:770–83. doi: 10.1360/TB-2021-0856

[33] Prasetyo HE Ramba Y Noyiana M. ABA vs brain gym for gross motor skills in autistic children. J Phys Conf Ser. (2020) 1529:032032. doi: 10.1088/1742-6596/1529/3/032032

[34] So WC Wong M Lam K. Social and communication skills predict imitation in ASD. Front Educ. (2016) 1:3. doi: 10.3389/feduc.2016.00003

[35] Zampella CJ Csumitta KD Simon E Bennetto L. Interactional synchrony and social ability in ASD. J Autism Dev Disord. (2020) 50:3195–206. doi: 10.1007/s10803-020-04412-8, 32065341 PMC7569722

[36] Chen J Cao M Jing J. Motor deficits in ASD children. Chin J Sch Health. (2020) 41:1590–4. doi: 10.16835/j.cnki.1000-9817

[37] Wang Z Zhang D He F Liang B Huang R Liu M. Biological motion perception in social cognition. Psychol Sci. (2014) 37:1055–9. doi: 10.16719/j.cnki.1671-6981

[38] Craig F Fanizza I Russo L Lucarelli E Alessandro L Pasca MG . DSM-5 vs ACSF:SC correlation in ASD social communication. Autism Res. (2017) 10:1249–58. doi: 10.1002/aur.1772, 28266789

